# Post-Partum Hæmorrhage

**Published:** 1910-07-23

**Authors:** G. E. Herman

**Affiliations:** Consulting Obstetric Physician, London Hospital.


### Hospital Clinics.

### POST-PARTUM HÆMORRHAGE.

By G. E. HERMAN, M.B., F.R.C.P., Consulting Obstetric Physician, London Hospital.

(Delivered at the Medical Graduates' College and Polyclinic, Chenies Street, W.C., on Monday, June 6, 1910.)

Gentlemen,—The subject of my lecture is post-partum hæmorrhage; hæmorrhage occurring during the third stage of labour. The third stage of labour consists in the delivery of the placenta; and in the natural third stage hæmorrhage is prevented by uterine contraction and retraction. These two things are not the same; you may have contraction without retraction, and retraction without contraction. Contraction of the uterus is a process like that of the contraction of voluntary muscles. Retraction means that the work in each contraction is not altogether undone; the uterus between contractions does not go back to the exact state in which it was before the contraction; the cavity remains smaller than it was before. In the third stage of labour you have the opportunity of observing retraction without contraction. If you put your hand on the uterus in the natural third stage of labour you find the uterus is about the size of a fœtal head; it is firm, not indefinite. And every now and then you can feel the uterus becoming as hard as a cricket ball. That is contraction; it passes off, and the uterus remains retracted. You have retraction with intermittent contraction. Occasionally—but it is rare—you get contraction without retraction. I have seen this after the induction of premature labour by Champetier's bag, and I have heard similar cases described by others; therefore I do not think it is so uncommon as it may be supposed to be. You may get the cervix dilated till its diameter is nearly four inches, and the uterus every now and then hardening, and then the hardening passing off. The fœtus remains above the pelvis freely movable, in spite of the intermittent contractions. There are contractions, but not retraction, and therefore the child does not advance. Uterine contraction is independent of the brain; it goes on after the spinal cord has been divided. In paraplegia a woman is delivered just as in an ordinary condition of health. It is independent of the spinal cord. The nerve force which enables contraction and retraction to take place is derived from the ganglia in the uterus itself. These contractions are like contractions produced elsewhere by the sympathetic nerves—not so sharp and violent as those produced by excitation of the grey matter of the brain or spinal cord; they come on and pass off gradually, and are intermittent. The reason of that is that when the contraction has passed off, the nerve force in the uterine ganglia is for the time exhausted, and you do not get a fresh contraction until that energy has been restored.

There are certain rare forms of post-partum hæmorrhage which are described in nearly all the text-books. They do occur, but they are so rare that I cannot recall having seen them. It is said there may be hæmorrhage into the peritoneum from rupture of varicose veins or from ruptured adhesions, also from severe lacerations of the cervix. And it is said you may get hæmorrhage from hæmophilia. Hæmophilia is handed on in the male line; seldom in the female line. It is said there may be hæmorrhage from Bright's disease. I have seen pregnancy and labour in connection with Bright's disease, in which there was no excessive hæmorrhage. Again, it has been said to occur from purpura. I grant that all these things may exist, but they are so rare that you can dismiss them from your mind as causes of post-partum hæmorrhage in the cases which you have to treat in practice. The common form of post-partum hæmorrhage comes from imperfect contraction and retraction of the uterus.

Why should not the uterus contract and retract properly? There are two main conditions which prevent this. The first is, when the uterus is not empty. If there is a piece of placenta retained that piece will mechanically prevent the uterus from properly contracting. If the placenta were everywhere adherent there would be no post-partum hæmorrhage; there would be nothing to bleed. But if a small part of the placenta is adherent under the influence of uterine contractions, part of the placenta may be torn off and expelled, and the adherent part remain behind. Then the uterus cannot properly retract and contract and check the hæmorrhage. It may be adherent placenta or it may be a placenta which, in consequence of hæmorrhage at an early period of pregnancy, has got separated from the rest of the placenta by tissue in which there are no chorionic villi. This is called a succenturiate placenta. Or it may be only a piece of chorion. When the placenta has been expelled it should always be examined. If there is a succenturiate placenta, and you examine the membranes, you will see that a bit of chorion is missing. But that is not so satisfactory as it sounds, because if there has been much manipulation of placenta and membranes it may be difficult to reconstruct the membranes as they were. Another evidence is noting a vessel running to the edge of the placenta and breaking short off. If you see that, you know those vessels must have gone to a piece of placenta.

The causes of retention of chorion are chiefly (*a*) too early rupture of membranes or (*b*) too late rupture of membranes. If the membranes remain entire for the proper time chorion and amnion can only advance together through the os uteri if the chorion has separated from the body of the uterus. (*a*) If the membranes rupture too early then the chorion is not separated, but remains adherent. (*b*) It is common to use the term "the child was born in its membranes." My experience is that it is very rare indeed for a child to be born in its membranes. What is meant is that it is born in its amnion. The amnion advances through the rent in the chorion, and the separation of the chorion from the uterus is incomplete, and there is retention of chorion. These are the chief conditions which prevent the uterus from being emptied. There is another which I might mention, and that is the presence of a fibroid which will prevent its proper contraction and retraction.

From the recollection of those facts there arise two broad therapeutic propositions. One is, the first thing in treatment is to make the uterus contract. If you cannot do that, the next thing is to find out why the uterus will not contract. You do that by putting the hand in the uterus to see if there is anything in the uterine cavity. If there is a fibroid, it may be enucleated. If there is placenta, that should be brought away; if it is adherent it must be detached. Exploration with the hand in the uterine cavity takes the second place to rubbing and kneading by the abdomen. A woman will not appreciate an accoucheur putting his hand into the uterus without good reason. And there is a little danger in doing so. The excitation of contraction and the emptying of the uterus will in the vast majority of cases be successful in stopping bleeding. It will always be successful where the uterine energy is not exhausted. It is a precept in text-books that the prevention of post-partum hæmorrhage consists in the proper management of the third stage of labour. Of course that is important; but of still more importance is the proper management of the second stage. That, in my judgment, is the vital point in the preventive treatment of post-partum hæmorrhage. You will find it sometimes stated that there are women who are especially liable to post-partum hæmorrhage, and who get it in confinement after confinement. I myself do not think that such persons exist. It means that there are some doctors who meet with post-partum hæmorrhage, and a woman who is attended by one of these doctors each time probably gets the hæmorrhage each time. But if she has a different doctor she may not get hæmorrhage again. I remember hearing a discussion once among a group of medical men, who were talking over the various ways in which they treated post-partum hæmorrhage. There was one man, rather deaf, who had been standing apart and taking no part in the discussion. One of the doctors turned to him and asked him how he treated post-partum hæmorrhage. His reply was very simple: "I have never had a case." I asked him as to his management of labour, and found that he was always most careful never to drag a child out when the uterus was not acting. We sometimes have to effect delivery when the child is large or the pelvis small, or both. When with forceps the child has been dragged through the pelvis it comes to bulge the perineum. By the time the head has got so low as to make the perineum bulge there is no longer any bony obstruction to its advance, and this is the time to take off the forceps and leave the head resting on the perineum. The pressure of the head there, if the uterus is not exhausted, will provoke enough uterine energy to drive it out.

In breech presentations it is a familiar and very important axiom to do nothing to hurry delivery until the child's body has been born. If you have acted on this precept, the uterus will not have become exhausted. Even then you should wait for the onset of a pain before extracting the head, and if the pain passes off before you can complete it wait until the next one. The same applies in delivery after craniotomy—namely, to pull only when a contraction is on. You are told to follow down the uterus in post-partum hæmorrhage. If you act on the precept I have just told you the uterus will take care of itself.

There are two therapeutic agents which have an effect on the uterus: one is ergot, which has been used for many years, and the other is pituitary extract. Each of these will make the uterus contract and remain contracted if the energy of the nervous ganglia of the uterus is not exhausted. If that energy is exhausted no drug will make it contract. Ergot will not; I do not know whether pituitary extract will—it is a new thing and I have made no observations on it. The great point in the management of the second stage of labour consists in not allowing the nervous energy of the uterus to get exhausted. If you carry out what I have said you may go through years of practice and never get a case of post-partum hæmorrhage. Still, you may be called to cases in which somebody else has not acted on this good rule, in which the patient is flooding seriously, and you must stop the hæmorrhage. The first thing is to get the uterus to contract. Obviously the best way is to manipulate it through the abdominal wall by rubbing and kneading. Then it will usually contract. Whether you will keep it contracted by this means is another matter. It is a good method, and as no instruments are required time is not lost in sterilising. If the contraction does not continue, the next thing is to put the hand in the uterus, to make sure that it is empty. It has been said, as a reason for putting the hand in the uterus, that it stimulates contraction; but though that may be theoretically good, it is not a sufficient reason, neither is this a sufficient preventive of further hæmorrhage, because a uterus cannot properly contract while your hand is inside it. The object of putting the hand in the uterus is to make sure that it is emptied and to remove anything that may be in its cavity. Having done these things, there is another mode of causing reflex stimulation, which is by injecting water as hot as the patient can bear it. There should be hot water in every lying-in room. It is not necessary to occupy time in taking the exact temperature of the water; put your hand into it, not merely the finger, because the finger can stand a higher temperature than the whole hand. A temperature which the hand can bear you may be sure the uterus will also bear. You must do it quickly, because if you delay the patient may be dead. Moreover, there may have been some oversight in the antiseptic precautions, and this use of hot water will cleanse the cavity and wash down decidua or clot which may have escaped your fingers. You may put ice into the uterus. But you cannot always hurry off to the fish-shop for ice. If you have used hot water and the uterus does not continue to contract, then the next thing to be done is to compress the uterus between both hands, one hand in the vagina and the other on the abdominal wall. Pressure of the uterus has been recommended in various ways. You can press the uterus against the symphisis pubis, or compress it against the spine. Another measure is to put the hand flat in the vagina and bend the neck of the uterus forward, so as to compress it between the flat hand and the outside. That is not altogether good, because if it is done effectually there is a kink in the canal where the uterus is bent, which will prevent the uterus being properly emptied if there is fluid still in it.

There are other remedies which I would like to class as remedies of the past, because I think they will not last. Bimanual compression of the uterus is so effective that I think it will hold its ground when other methods have fallen into disuse. There used to be a plan, owing its place to the energy and fluency of the late Dr. Robert Barnes, of injecting perchloride of iron into the uterus, the object being to clot the blood and so mechanically stop the hæmorrhage. That is not an infallible remedy, because there have been a fair number of cases—I do not say many, because probably it has not been used often—in which the injection failed to stop the hæmorrhage. But it is bad, because it leaves the uterus in an unsatisfactory condition. Perchloride of iron kills the blood, and so after the injection the uterus is left filled with a mass of dead clot, which clot often decomposes, and the patient may be ill afterwards with sapræmia. Cases have occurred in which the patient was supposed to have died from embolism owing to clots being carried into the circulation; but I think that is probably problematical. Still, a clot is a suitable nidus for saprophylic organisms.

There is another method of treatment, introduced more recently, which I suppose has replaced perchloride of iron amongst those who do not manage the second stage of labour properly—namely, plugging the uterus with iodoform gauze. Post-partum hæmorrhage seldom causes death; I have not the figures with me, but it is a very rare cause in lying-in charities. When you get a gentleman who in the space of a year or two has accumulated a hundred cases in which he plugged with gauze, it is impossible to avoid the conclusion that most of them would have done as well if they had not been plugged; indeed, rather better. Plugging with iodoform gauze is better than filling the uterus with perchloride of iron, because, when it is taken out, the uterus is left fairly clean.

There is another objection, that to be prepared with the gauze necessitates carrying a larger bag about; the gauze requires a hermetically sealed tin, to make sure that it is sterile.

Another remedy has lately been much talked about, and that is compression of the abdominal aorta. It is possible so to compress that vessel that no blood shall go from it to the uterus or the lower limbs. But how long that can be kept up I do not know. You must compress hard to be sure that you are stopping the blood current, and if it is kept up until the uterus is firmly contracted and retracted, it may require to be done for some time, and it is very fatiguing. Other people have used means to do this in a way independent of fatigue to the operator. One of the latest is encircling the body between the innominate bones and the ribs with a strong indiarubber tube, putting a pad over the aorta, and strongly fastening the ligature so that it may encircle the abdomen so tightly, and the pad press on the aorta so firmly, as to stop the supply of blood to the lower parts of the body. This has been done on the Continent for different operations, such as those on the hip joint, and the result has been that the operation has been carried out almost bloodlessly. But that leads back to the difficulty of carrying these things about; you need yards of indiarubber tubing, and it must be strong tubing, and recent, because indiarubber soon perishes, especially in hot countries. Moreover, if you use these means to cut off the blood-supply from a large area of the body, you necessarily increase the blood pressure in the remaining part. In experiments on animals there have been cases in which the application of a ligature sufficiently tight to press on the abdominal aorta has been followed by injuries to the viscera. I do not think these objections to it are of great moment in labour, because if the patient is dying from hæmorrhage, a little acceleration of the pulse is not a great matter, and injuries to the viscera are not common in puerperal women. I have not heard of cases in which it has resulted in harm. Still, I think most surgeons would prefer to stop hæmorrhage by pressure on the bleeding point. I have seen in a German book a suggestion that the operator should compress the abdominal aorta by putting a pad, consisting of 8 lb. of wet sand, on the abdomen. If you will tell me in how many homes you are likely to find 8 lb. of wet sand ready, I will begin to tell you whether I think there is any utility in it.

There is one more point about the treatment of post-partum hæmorrhage: If you have a patient who has lost a great deal of blood, you have stopped the bleeding, and the uterus is retracted and contracted, it does not follow that the patient is out of danger; because when she has lost much blood the effect upon her depends not upon the depletion of the number of red corpuscles, but on the loss of fluid from the vessels, fluid which should stimulate the heart to contract. And so the patient may die from exhaustion some hours after the hæmorrhage has all ceased. In such cases the indication of danger is that the patient is restless. If, when the hæmorrhage is stopped, the patient lies quietly and seems inclined to go to sleep, you may feel safe in leaving her. But if she is restless, tossing about in the bed, she is in danger, and the only treatment is to put more fluid into the blood vessels.

There are two ways of doing that, by injecting fluid into the cellular tissue, and by injecting into a vein. Injecting blood is an operation which is, or ought to be, extinct, because it is a danger. The fluid to use is that made by putting a teaspoonful of salt into a pint of water. The water should have been boiled, and the salt should be clean. Boiling takes time, but there should always be boiling water in the house of a lying-in woman. You can inject a couple of pints without causing the patient danger. A case occurred in the London Hospital in which the Resident Accoucheur injected plain water, and no harm resulted. The object of adding the saline is to make the fluid of the specific gravity of the blood. Injection into a vein is better because it is quicker. You may not have the instruments with you, and if you are not experienced in dissection you may have some difficulty in finding the vein. In such a case you can inject fluid into the cellular tissue. This can be done on the plan followed by my late colleague, Mr. Barnard, of putting fluid in a jug about a foot above the level of the patient's body, and letting the saline flow in at the rate of about a pint an hour. There is no advantage in injecting so quickly as to produce a painful swelling. There is another way in which you can get fluid quickly into the circulation, and that is by injecting it into the bowel. The essential points are that the fluid should be injected slowly, and let run in as high up as possible.

But I repeat that the essential treatment of post-partum hæmorrhage is the preventive treatment, and the preventive treatment consists in the proper management of the *second* stage of labour.

